# Advancing Biomarker Research in Depression: The role of neuroprotective and inflammatory markers

**DOI:** 10.1192/j.eurpsy.2025.164

**Published:** 2025-08-26

**Authors:** J. Eder, P. Falkai, POKAL Group

**Affiliations:** Department of Psychiatry and Psychotherapy, LMU University Hospital, Munich, Germany

## Abstract

**Abstract:**

The search for biomarkers to diagnose depression is an endeavor being pursued in psychiatric research since the 1980s, but hasn’t resulted in clinical application. This remains challenging since the symptomatology of depression is very heterogeneous and because there is no diagnostic gold standard that focuses on an underlying biological mechanism. Despite knowing about the metabolic, endocrine, inflammatory as well as autonomous dysregulation that has been observed in depressed patients, none of these are broadly used to stratify patients. A novel area of research involves the insulin-like growth factor (IGF) system, which plays a vital role in brain development, neurogenesis, and neuroprotection. The insulin-like growth factor (IGF) system, encompassing IGF-I, IGF-II, IGFBPs (1-6), and their receptors, is critical for brain development, neurogenesis, neuroplasticity, and neuroprotection. Insulin-like growth factor binding protein-2 (IGFBP-2), the predominant IGFBP in the central nervous system, regulates IGF-I and IGF-II bioavailability, half-life, localization, and receptor interactions. Serum levels of IGFBP-2 inversely correlate with DTI-derived myelin integrity measures, especially in anterior brain regions.

In a data-driven clustering analysis of a depressed cohort, elevated IGFBP-2 levels delineated a healthier subgroup within a hospitalized cohort of patients with unipolar depression. Additionally we discovered, that patients with higher IGFBP2 levels at inclusion were more likely to remit faster concerning their depressive symptoms, in contrast to an inflammatory marker-defined subgroup. These findings suggest IGFBP-2 as a biomarker for stratifying patients and tailoring interventions in depression. Future research should explore IGFBP-2 and inflammatory markers to better stratify patients and develop targeted therapies, advancing precision medicine for depression and related disorders.

**Disclosure of Interest:**

J. Eder: None Declared, P. Falkai Consultant of: Peter Falkai is on the advisory boards of Janssen, Lundbeck, Otsuka, Servier, and Richter, Speakers bureau of: Peter Falkai receives speaker fees from Janssen, Lundbeck, Otsuka, Servier, and Richter

